# Origin and functional role of antisense transcription in endogenous and exogenous retroviruses

**DOI:** 10.1186/s12977-023-00622-x

**Published:** 2023-05-16

**Authors:** Fabio Romerio

**Affiliations:** Department of Molecular and Comparative Pathobiology, Johns Hopkins University School of Medicine, Baltimore, MD USA

## Abstract

Most proteins expressed by endogenous and exogenous retroviruses are encoded in the sense (positive) strand of the genome and are under the control of regulatory elements within the 5’ long terminal repeat (LTR). A number of retroviral genomes also encode genes in the antisense (negative) strand and their expression is under the control of negative sense promoters within the 3’ LTR. In the case of the Human T-cell Lymphotropic Virus 1 (HTLV-1), the antisense protein HBZ has been shown to play a critical role in the virus lifecycle and in the pathogenic process, while the function of the Human Immunodeficiency Virus 1 (HIV-1) antisense protein ASP remains unknown. However, the expression of 3’ LTR-driven antisense transcripts is not always demonstrably associated with the presence of an antisense open reading frame encoding a viral protein. Moreover, even in the case of retroviruses that do express an antisense protein, such as HTLV-1 and the pandemic strains of HIV-1, the 3’ LTR-driven antisense transcript shows both protein-coding and noncoding activities. Indeed, the ability to express antisense transcripts appears to be phylogenetically more widespread among endogenous and exogenous retroviruses than the presence of a functional antisense open reading frame within these transcripts. This suggests that retroviral antisense transcripts may have originated as noncoding molecules with regulatory activity that in some cases later acquired protein-coding function. Here, we will review examples of endogenous and exogenous retroviral antisense transcripts, and the ways through which they benefit viral persistence in the host.

## Introduction

Retroviruses (*Retroviridae*) are a family of positive-sense single stranded RNA (ssRNA) viruses that infect vertebrates [1]. They are classified into two subfamilies (*Orthoretrovirinae* and *Spumavirinae*) and several *genera*. The defining feature of retroviruses is that they encode a reverse transcriptase (RT, an RNA- and DNA-dependent DNA polymerase) that converts the ssRNA genome into a dsDNA molecule, and an integrase (IN) that inserts the dsDNA molecule into the host genome.

All retroviral genomes include five essential genetic elements: two identical long terminal repeats (LTR), and three essential genes: the structural genes *gag* and *env*, and the enzymatic genes *pol/pro*. The two LTRs are identical direct repeat sequences located at the 5’ and 3’ ends of the genome, they are arranged in a tail-to-head orientation and have regulatory roles: the 5’ LTR contains the promoter driving expression of the viral genes, and the 3’ LTR contains the polyadenylation signal. Retroviral *genera* that contain only these five elements (alpha-, beta-, and gammaretroviruses) are said to have a “simple” genome. On the other hand, deltaretroviruses, epsilonretroviruses, lentiviruses, and all spumaviruses encode additional regulatory and accessory proteins, and thus have a “complex” genome. In many cases, these genes overlap each other and are encoded in different reading frames. Their expression involves two or three classes of transcripts produced from a single RNA molecule encompassing the entire viral genome: full length and singly spliced transcripts for retroviruses with simple genome, plus multiply spliced transcripts for retroviruses with complex genomes [1].

Over millions of years, now-extinct ancestral exogenous retroviruses infected and colonized their hosts and persist to this day as endogenous retroviruses (ERV) [2–4]. While present-day exogenous retroviruses only infect somatic cells, ancestral forms may have also been capable of infecting cells of the germline, which ensured that they may be transmitted vertically and become fixed in the population [4, 5]. Today, ERVs occupy a large fraction of vertebrate genomes. In humans, endogenous retroviruses (HERV) account for ~ 8% of the genome with ~ 700,000 *loci* [6, 7]. HERVs are broadly grouped into Class I (similar to gamma- and epsilonretroviruses, Class II (similar to betaretroviruses), and Class III (similar to spumaviruses). While most ERVs present a simple genome that includes the five basic genetic elements [4], some (such as HERV-K) can under certain circumstances express additional proteins [8, 9].

### Retroviral LTRs as bidirectional promoters

The retroviral 5’ and 3’ LTRs play regulatory roles in viral expression. Specifically, the U3 region of the 5’ LTR – organized in promoter, enhancer, and modulatory domains – contains binding motifs for a wide array of transcription factors that promote and regulate expression of the viral genes. Retroviruses such as HTLV-1 and HIV-1 also express regulatory proteins that function as transactivators and augment transcription from their own 5’ LTR through positive-feedback loops. The HTLV-1 Tax protein promotes transcription by recruiting to the 5’ LTR transcription factors of the ATF/CREB family as well as p300/CBP [10]. The HIV-1 Tat protein recruits P-TEFb to the 5’ LTR, which in turn increases processivity of RNA polymerase II and promotes transcription elongation [11]. At the other end of the proviral genome, the 3’ LTR contains the motifs required for polyadenylation of the pre-mRNA, which ensures its proper processing, nuclear export, stability, and translation [12].

As discussed above, 5’ and 3’ LTRs are identical direct repeats and contain the same transcriptional regulatory elements. While the presence of a polyadenylation signal in the 3’ LTR is essential to proper processing the viral transcripts, the presence of a polyadenylation signal in the 5’ LTR could impact viral expression. The location of the polyadenylation signals separates retroviruses into two groups [13]. On one hand, viruses such as HTLV-1, HTLV-2, BLV, RSV, and MMTV use bipartite polyadenylations signals: an AAUAAA sequence (recognized by the cleavage and polyadenylation specific factor, CPSF) located in the U3 region upstream of the transcription start site (U3-R boundary), and a GU/U-rich sequence (recognized by the cleavage stimulation factor, CstF) positioned in the beginning of the U5 region. Therefore, the full polyadenylation signal is present only at the 3’ end of the viral transcripts (3’ LTR). Further, in RSV and MMTV, the R region is very short, thus keeping the two signals at a functional distance. However, in HTLV-1, the R region is 275 bp, and functional proximity of the two polyadenylation signals is achieved via secondary structure (looping) of the viral transcript [13]. In the second group of retroviruses (e.g., HIV-1, HIV-2, EIAV, MoMLV) both polyadenylation signals (AAUAAA and GU/U-rich sequences) are located in the R region, and therefore are transcribed at both ends of the viral RNA molecule. In the case of HIV-1, two mechanisms ensure utilization exclusively of the polyadenylation signals at the 3’ end of the transcripts: the presence of U3-derived sequences with “polyadenylation enhancer” activity (which are not present at the 5’ end) [14–17], and the formation of secondary structures that suppress the polyadenylation activity of the signals at the 5’ end of the transcript [18–20]. Suppression of the 5’ polyadenylation signal has also been shown to involve binding the of U1 snRNP splicing factor to the major splice donor site at the 5’ end of the viral RNA [14, 21, 22].

Several studies have provided mounting evidence that retroviral LTRs are capable of bidirectional transcription. An early report by Larocca and colleagues showed that the 3’ LTR of HTLV-1 can direct antisense transcription in a Tax-independent manner [23]. A more recent study showed that sense and antisense transcription across the HTLV-1 provirus do not interfere with each other and with the expression of Tax or the HTLV-1 antisense protein, HBZ [24]. The same study also showed that, in the absence of Tax, antisense transcription predominates [24]. A negative sense promoter has also been identified within the 3’ LTR of HIV-1 [25–27]. Antisense transcription driven by the LTR of both HTLV-1 and HIV-1 is independent of – or inhibited by – their respective transactivators, Tax and Tat [25–27]. Interestingly, the ability to drive bidirectional transcription is not limited to complex retroviruses. Indeed, the LTRs of the simple retrovirus, murine leukemia virus (MLV) and also endogenous retroviruses can direct sense and antisense transcription [26, 28–31].

Altogether, bidirectional transcriptional activity is widespread among simple and complex exogenous retroviruses as well as endogenous retroviruses. This suggests that this property has an ancestral origin, it has been conserved in present-day retroviruses, and thus it serves a purpose that benefits viral persistence or spread.

### Antisense transcription in exogenous retroviruses

**Fig. 1:**
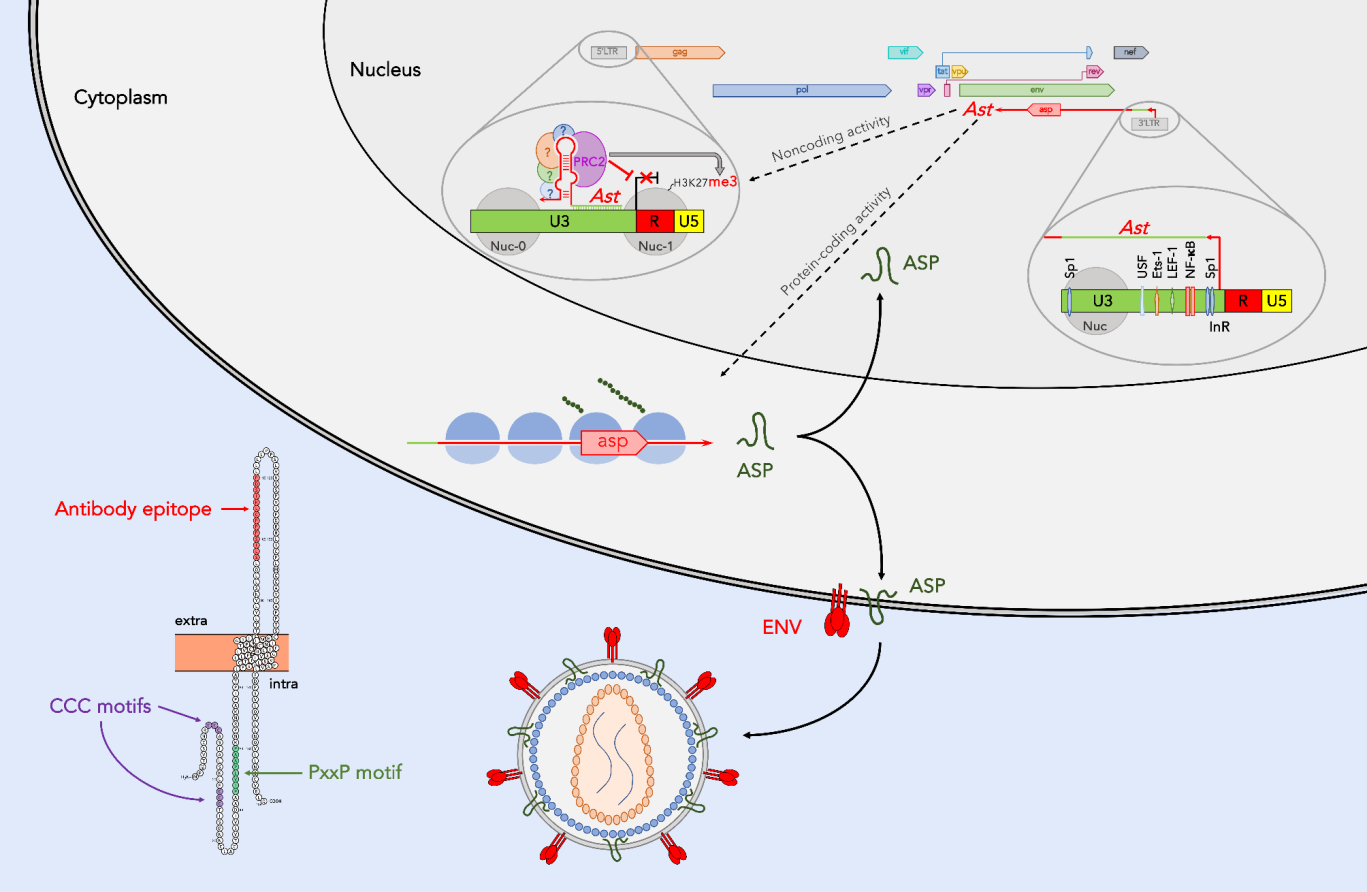
The HIV-1 antisense gene, *asp*. The figure shows a schematic representation of the HIV-1 proviral genome with structural and enzymatic genes (*gag, pol, env*), regulatory genes (*tat* and *rev*), and accessory genes (*vif, vpr, vpu, nef*) expressed from the proviral 5’ LTR. The antisense gene *asp* is expressed from a negative sense promoter in the U3 region of the 3’ LTR in a manner independent of the viral transactivator, Tat. The negative sense promoter contains binding sites for USF, Ets-1, LEF-1, Sp1 and NF-κB. The antisense transcript *Ast* is a bifunctional RNA with both noncoding and protein-coding activities. The former is carried out in the nucleus: *Ast* acts as a lncRNA that promotes epigenetic silencing of HIV-1 by recruiting the histone methyltransferase (PRC2) to the 5’ LTR leading to trimethylation of lysine 27 on histone H3 (H3K27me3), which leads to assembly of the nucleosome Nuc-1, and inhibition of transcription. In addition, *Ast* is translocated to the cytoplasm where it functions as a mRNA and leads to the expression of the antisense protein ASP. In non-productively infected cells, ASP accumulates in the nucleus, whereas in productively infected cells ASP localizes in the cytoplasm and on the cell membrane in close proximity of the ENV. Further, upon viral budding and release, ASP is also detectable on the viral envelope

*HIV-1 and other lentiviruses.* The first retroviral antisense gene to be described was the antisense protein (*asp*) gene of HIV-1 [32]. The *asp* ORF was identified through sequence analysis of twelve HIV-1 viral isolates, and it maps in the same genomic region as the *env* gene straddling the gp120/gp41 junction (Fig. 1). The product of this gene – the antisense protein (ASP) – is a polypeptide of ~ 190 residues with high content of hydrophobic amino acids, which suggests an association with cellular membranes [32]. While the original study did not include experimental evidence that the *asp* ORF encodes an actual protein, it provided clues in support of that conclusion. First was the evidence that the ORF is longer than 100 codons, which is uncommon in DNA strands complementary to known genes [33]. Second, the sequences analyzed showed the presence of signals necessary for production of an antisense mRNA transcript, such as the promoter, poly-A addition signal and site, and the downstream G and T domains. Finally, conserved sequences necessary for protein translation were also detected, including canonical *start* and *stop* codons, and a codon periodicity of ‘G-nonG-N’ [32].

Experimental evidence of antisense transcription in the HIV-1 genome first came in 1990 through the use of Northern blot analysis of poly-A + RNA extracted from acutely infected cells [34] (Table 1). Subsequently, the use of RT-PCR allowed to prove HIV-1 antisense transcription in chronically infected T- and myeloid-derived cell lines, and also in clinical samples from early-stage, asymptomatic people living with HIV-1 (PLWH) [25, 35]. Further, the introduction of strand-specific RT-qPCR assays able to avoid artifacts due to endogenous and/or self-priming provided stronger evidence of antisense transcription in the HIV-1 proviral genome [36–38]. Antisense transcription was also detected in studies employing high-throughput sequencing methods [39].

**Table 1:** Antisense transcription activity in endogenous and exogenous retroviruses

Retrovirus	*Genus*	Species	Bidirectional LTR	Antisense transcript	Distribution and function of transcript	Antisense protein	Distribution and function of protein
Exogenous	Gamma-	MLV	**P**	?	?	?	?
	Delta-	HTLV-1	**P**	*Hbz*	Primarily nucleus Promotes cell survival and proliferation Encodes HBZ	HBZ	Nucleus Promotes viral latency and persistence
		HTLV-2	**P**	*Aph-2*	Unknown distribution Encodes APH-2	APH-2	Nucleus Inhibits viral replication
		HTLV-3	**P**	*Aph-3*	?	APH-3	Nucleus, cytoplasm Inhibits LTR activity
		HTLV-4	**P**	*Aph-4*	?	APH-4	Nucleus Inhibits LTR activity
		STLV	**P**	*Sbz*	?	SBZ	Nucleus Promotes viral latency and persistence
		BLV	**P**	*AS1, AS2*	Nucleus Promote cell proliferation	**O**	**O**
	Lentiviruses	HIV-1 (M)	**P**	*Ast*, ASP RNA	Primarily nucleus Promotes latency Encodes ASP	ASP	Nucleus, cytoplasm, cell surface, viral envelope Unknown role
		FIV	**P**	**P**	?	**O**	**O**
		BIV	**P**	**P**	?	**O**	**O**
Endogenous	Class I	HERV1	**P**	?	?	**O**	**O**
		HERV9	**P**	?	?	**O**	**O**
	Class II	HERV-K	**P**	**P**	?	**O**	**O**
		muIAP	**P**	**P**	?	**O**	**O**
	Class III	muERV-L	**P**	**P**	?	**O**	**O**

Gamma- = Gammaretroviruses; Delta- = Deltraretroviruses; ? = unknown, not reported; **P** = reported, confirmed; **O** = absent, not found

The structure of the HIV-1 antisense transcripts and the mechanisms that regulate the expression were the focus of several reports. The earliest identified an antisense RNA of 2242 nt that originated in the R region of the 3’ LTR and terminated in a poly-A tract [25]. These results were confirmed by a later report [40]. However, Landry et al. identified multiple transcription start sites in the U3 region as well as in the *nef* and *env* genes, and also a polyadenylation signal in the *pol* gene [36]. A more in-depth analysis by Kobayashi-Ishihara and colleagues described a major antisense transcript (*ASP-L* or *Ast*) of 2574 nt with start site in the U3 region of the 3’LTR and a termination site in the *env* gene (Fig. 1) [37]. Interestingly, the same study demonstrated that a large fraction of HIV-1 antisense transcripts has a predominantly nuclear localization [37].

The location of the 5’ terminus of the antisense transcripts suggested that their expression is directed by a negative sense promoter (NSP) within the 3’LTR [25], which was shown to have 3- to 9-fold lower activity than that of the HIV-1 positive sense promoter (PSP), and it was inhibited by Tat expression, possibly by directing the transcriptional machinery to the PSP [25, 27]. The report by Michael et al. showed that NSP is a TATA-less promoter, and that the NF-κB and USF binding sites are critical for its activity [25], and a subsequent report identified an Sp1 binding site that is essential for NSP function [41]. Bentley et al. described regions of the 3’LTR with moderate, profound, and variable impact on NSP activity [27]. The segment of the 3’LTR with profound impact on NSP activity was mapped in the U3 region, and it contains binding sites for Sp1, NF-κB, LEF-1, Ets-1, and USF (Fig. 1) [27]. Disruption of the TATA box in the positive strand of the U3 region in the 3’ LTR increased NSP activity, which supports the notion that NSP is a TATA-less promoter and suggests that antisense transcription is under the control of an initiator element (InR) [27, 37]. Indeed, two putative InRs were later identified within the U3 region of the 3’ LTR [42], and a third one within in the R region (Fig. 1) [40]. A recent study from the Matsuoka group showed that HIV-1 antisense transcripts are inefficiently polyadenylated, which promotes their nuclear retention [43]. Our group investigated additional mechanisms involved in regulating the expression and possibly the function of HIV-1 antisense transcripts. First, we reported that the activity of the NSP within the HIV-1 3’ LTR is under epigenetic regulation [44]. In particular, we found the presence of a nucleosome over the U3 region and in close proximity of the *nef*-3’ LTR boundary (Fig. 1). Assembly and disassembly of this nucleosome is under the control of epigenetic modifications of lysine 9 and 27 on histone H3: acetylation of these residues increases transcriptional activity of NSP and promotes antisense transcription, whereas di- and trimethylation of these residues has the opposite effects [44]. In addition, we identified and precisely mapped post-transcriptional (epigenetic) base modifications deposited on HIV-1 antisense transcripts, which include primarily ribose methylation at multiple adenosine and guanosine residues, and pseudouridylation [45]. Studies are underway that seek to address whether dynamic addition/removal of these modification contributes to regulate the stability, sub-cellular localization, interaction with binding partners, and functional activity of HIV-1 antisense transcripts.

In line with that, studies from several groups including our own have shown that HIV-1 antisense transcripts act as bifunctional RNAs (Fig. 1). In addition to serving as mRNA for the expression of the HIV-1 antisense protein ASP, these transcripts also function as noncoding RNAs that regulate the expression of HIV-1 sense transcripts. An early report showed HIV-1 antisense transcripts reduce the expression HIV-1 *Gag* RNA, the levels of HIV-1 proviral DNA, and viral production in the culture supernatant [37]. Subsequently, Kevin Morris’ group provided evidence that HIV-1 antisense transcripts promote HIV-1 latency via epigenetic silencing of HIV-1 transcription [46]. In that report, Saayman et al. showed that knockdown of HIV-1 antisense transcripts resulted in a reduction in suppressive epigenetic marks (H3K9me2 and H3K27me3) at the 5’LTR, and they also demonstrated that HIV-1 antisense transcripts interact with the DNA methyltransferases [46]. Our group reported that ectopic overexpression of HIV-1 antisense transcripts lacking protein-coding capacity suppressed basal HIV-1 transcription during latency, inhibited latency reversal, and accelerated re-establishment of latency [38]. In addition, overexpression of HIV-1 antisense RNA maintained high levels of PRC2 and the suppressive epigenetic mark H3K27me3 at the HIV-1 5’LTR even after treatment with LRAs. We also showed that these effects involve interaction with subunits of the epigenetic silencer PRC2 (Fig. 1) [38]. A more in-depth discussion can be found in a recent review by Li et al. [47].

Sequence analyses and computer modeling suggest that the protein encoded by HIV-1 antisense transcripts (ASP) includes intracellular N- and C-termini, two transmembrane domains and an intervening extracellular loop [48, 49]. Additional features of interest are two closely spaced cysteine triplets in the N-terminal portion of the protein and a highly conserved PxxPxxP motif located between residues 40–50 of the protein (Fig. 1). Thorough and systematic experimental analyses are needed to assess the functional role of these and possibly other yet unidentified ASP domains [50]. A very recent study utilized molecular modeling and dynamics simulation to predict the 3D-structures of ASP [51]. The *in silico* analyses described in that study identified three possible functional domains in ASP, namely the Von Willebrand Factor Domain-A (VWFA), the Integrin subunit alpha-X (ITGSX), and the ETV6-Transcriptional repressor [51]. Wet lab molecular studies are required to confirm the validity of these findings, and to ascertain the role these domains play in the mechanisms of action of ASP. Despite being first identified more than 30 years ago, the role of ASP in the virus lifecycle remains largely a mystery, which is in part due to its low expression levels. Additionally, the hydrophobic properties of ASP make it exceptionally challenging to raise antibodies able to reliably detect it. Nevertheless, expression of ASP has been demonstrated in several cell systems. An early study used electron microscopy to show that ASP associates with plasma, mitochondrial and nuclear membranes [139]. A later report found that ASP localizes to the plasma membrane (with both polarized and unpolarized expression patterns) as well as with cell surface protrusion [49]. More recently, we used flow cytometry and confocal microscopy to study ASP expression in several chronically infected lymphoid and myeloid cell lines and also in acutely infected primary human CD4 + T cells and monocyte-derived macrophages (MDM) [48]. Using a mouse monoclonal antibody against an epitope mapping in the extracellular loop of ASP, we found that ASP displays a polarized nuclear distribution in unstimulated, non-productively infected lymphoid and myeloid cell lines [48]. After cell stimulation and reactivation of productive infection, ASP was transported into the cytoplasm and to the plasma membrane where it colocalizes with the HIV-1 envelope glycoprotein ENV (Fig. 1). We also showed that upon budding and release from infected cells, ASP is present on the viral envelope (Fig. 1) [48].

The role of ASP in viral replication has been explored to a much lesser degree. Clerc and colleagues generated HIV-1 molecular clones with a premature *stop* codon in the *asp* ORF, but they were not able to show any appreciable difference in viral replication compared to the wildtype clone [49]. Two reports by the Barbeau group showed that ASP expression can induce autophagy in infected cells [52, 53]. Many viruses utilize autophagy to their advantage during their replication cycle [54]. In the case of HIV-1, autophagy is required for GAG processing during infection of macrophages, and it significantly increases viral production [55]. Therefore, these findings provide at least one possible role of ASP in HIV-1 replication.

Antisense transcription has also been shown to occur in other lentiviruses, including feline immunodeficiency virus (FIV) and bovine immunodeficiency virus (BIV) [56, 57] (Table 1). However, these and other lentiviruses do not express antisense proteins. Indeed, while a full-length *asp* ORF is present in the majority of HIV-1 strains of group M (responsible for the pandemic), it is absent in HIV-1 strains that belong to non-pandemic groups N, O, and P [58, 59]. Moreover, the frequency of a full-length *asp* ORF among strains of each group-M HIV-1 clade correlates with the prevalence of the clade [59]. The *asp* ORF is also absent in HIV-2 as well as in all species of lentiviruses infecting non-human primates (SIV infecting *Cercopithecidae* and African apes) [58, 59]. While many of these viral strains contain the canonical *start* and *stop* codons of the *asp* ORF, they also display one or more premature *stops* that prevent expression of a full-length ASP.

This suggests that the *asp* ORF originated recently, when group M diverged from its most immediate progenitor, SIVcpz_Ptt. This is supported by evidence we reported recently showing that the average number of internal *stop* codons in the genomic region of the *asp* ORF steadily declines in viral strains that are progressively closer to HIV-1 group M [58]. It also suggests the possibility that the creation and conservation of a full-length may facilitate viral spread or pathogenesis. On the other hand, this leaves open the question about the role that antisense transcription plays in the context of lentiviruses that do not express an antisense protein.

**Fig. 2:**
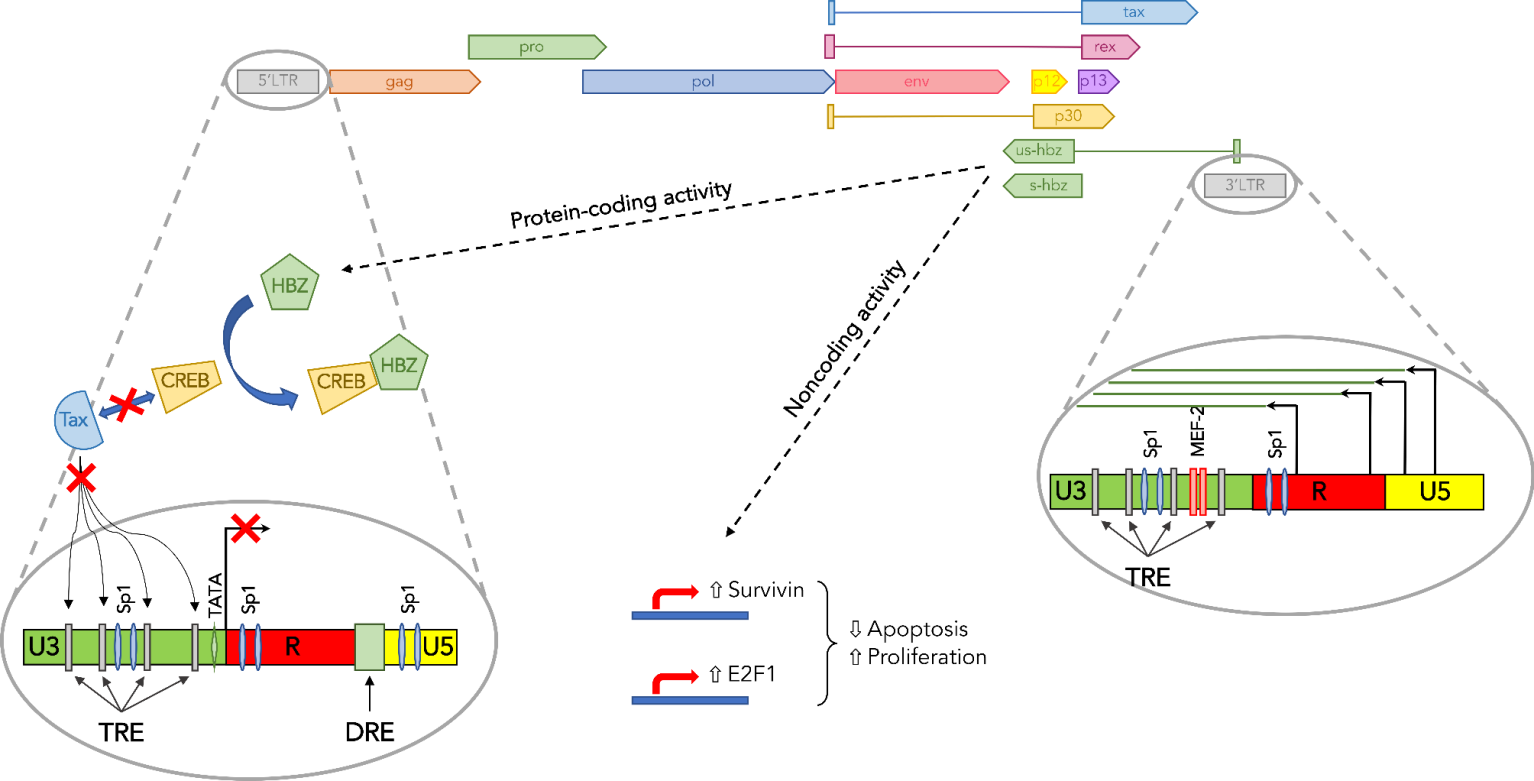
The HTLV-1 antisense gene, *hbz*. The figure shows a schematic representation of the HTLV-1 proviral genome with structural and enzymatic genes (*gag, pol/pro, env*), regulatory genes (*tax* and *rex*), and accessory genes (*p12, p13, p30*) expressed from the proviral 5’ LTR. The antisense gene *hbz* is expressed from the proviral 3’ LTRs in a manner independent of the viral transactivator, Tax. The *hbz* promoter contains binding sites for Sp1 and MEF-2. HTLV-1 produces spliced (s-hbz) and unspliced (us-hbz) antisense RNA variants, which encode two forms of the HBZ protein. *Hbz* transcripts have both noncoding and protein-coding activities. They act as lncRNAs that increase expression of Survivin and E2F1, which promote cell survival and proliferation, respectively. In addition, *Hbz* transcripts act as mRNAs that encode for the HBZ protein. HBZ interacts with CREB-2, it prevents the formation of the CREB-2/Tax heterodimer and binding to TRE sites in the HTLV-1 5’ LTR, and ultimately it downregulates HTLV-1 expression

*HTLV-1 and other deltaretroviruses.* Although evidence supporting the presence of antisense genes in the HIV-1 and HTLV-1 genomes came around the same time [23, 25, 34], much more progress has been made in understanding the role of the HTLV-1 antisense gene. Sense transcription of the HTLV-1 proviral genome is driven by the 5’ LTR. The HTLV-1 Tax transactivator induces sense transcription by interacting with phosphorylated CREB-2 when bound to Tax Response Elements (TRE) in the 5’ LTR (Fig. 2), and by recruiting the CBP/p300 coactivator [60]. Antisense transcription of HTLV-1 was shown to be driven by a TATA-less negative sense promoter in the proviral 3’ LTR that relies on Sp1 binding sites [23, 26, 61]. In addition to Sp1, the transcription factor MEF-2 has also been shown to promote HTLV-1 antisense transcription [62], which was found to start from multiple positions in the R and U5 regions of the 3’ LTR (Fig. 2) [63]. As reported for HIV-1, antisense transcripts of HTLV-1 show primarily nuclear distribution due to inefficient polyadenylation [43].

Demonstration that HTLV-1 encodes an antisense protein came in 2002 in a seminal paper by Jean Michel Mesnard and colleagues [64]. In their search for novel CREB-2 binding partners, the authors screened a cDNA library from the HTLV-1 infected MT2 cell line and isolated the cDNA of a previously unknown protein encoded in the negative strand of HTLV-1. Sequence analyses showed that the cDNA encodes a 31-kDa protein with features typical of bZIP transcription factors, hence its name: HTLV-1 bZIP factor, or HBZ [64]. Subsequent reports demonstrated that HTLV-1 also expresses both spliced and unspliced forms of the *Hbz* RNA (Fig. 2) [65–67], which differ in their 5′ untranslated regions and in the 5′ region of coding sequences resulting in slightly different N-terminal sequence of the two protein products (for a comprehensive review, see [68]). The protein product of the spliced *Hbz* RNA is expressed at higher levels than the product of the unspliced mRNA, therefore most studies have focused on the former (Table 1).

In their seminal report, Gaudray and colleagues showed that HBZ displays nuclear localization, that it interacts with CREB-2, and that it prevents binding of CREB-2 and Tax to TRE sites in the HTLV-1 5’ LTR, which results in downregulation of HTLV-1 expression (Fig. 2) [64]. Interaction of HBZ with CREB-2 and other members of the AP1 superfamily including c-Jun, JunB, JunD, CREB, MafB, and ATF3 was shown to require the bZIP domain in the C-terminus of HBZ [64, 69–75]. The N-terminal portion of the protein contains an activation domain that recruits the transcriptional coactivator CBP/p300 [64]. In addition to downregulating HTLV-1 expression, HBZ was shown to have a number of other properties. HBZ contributes to HTLV-1 latency by inhibiting the effects of Tax on activation of the canonical NF-κB pathway, which causes cell senescence [76, 77], and also by reducing Rex-mediated nuclear export of HTLV-1 unspliced or partially spliced transcripts [78]. HBZ has also been shown to promote cell proliferation via its interaction with members of the AP1 superfamily, ATF3 and JunD [69, 75]. The effects of HBZ on cell proliferation also involve its ability to induce the noncanonical Wtn ligand Wtn5 [79], and to upregulate expression of brain-derived neurotropic factor (BDNF) that promotes proliferation of leukemia cells [80]. HBZ reduces apoptosis and autophagy by inhibiting the expression of the pro-apoptotic molecule, Bim [81] and by inducing the mTOR pathway [82]. Additionally, HBZ affects genomic integrity [83] and telomere length [84], and it promotes inflammation [85–87]. Therefore, HBZ contributes to HTLV-1 persistence through multiple mechanisms. The simian counterpart of HTLV-1 (STLV-1) was shown to express spliced sense and antisense transcripts that encode Tax and SBZ (STLV-1 bZIP) proteins with functions very similar to the HTLV-1 factors [88] (Table 1).

As observed in the case of HIV-1, the antisense transcript of HTLV-1 is also bifunctional and has activities that are independent of its protein-coding role. Indeed, the *HBZ* RNA was shown to promote cell proliferation by increasing expression of E2F1 [66]. Moreover, the HTLV-1 antisense transcript has anti-apoptotic activities that involve increased expression of Survivin (Fig. 2) [89].

Among other members of the Deltaretrovirus *genus*, the human T-lymphotropic viruses 2, 3, and 4 (HTLV-2, 3, and 4) express antisense proteins called APH-2, 3, and 4 [90–92] (Table 1), although their role in viral replication is not as well characterized. HTLV-2 expresses a spliced antisense transcript from several start sites in the 3’ LTR, which encodes APH-2 [63, 93]. This protein lacks the bZIP domain found in HBZ, but can interact with the Tax protein of HTLV-2 (Tax2) and with CREB [94]. APH-2 shows nuclear, but not nucleolar, distribution. Similarly, HTLV-3 and 4 express spliced antisense transcripts that encode the APH-3 and APH-4 proteins, respectively [91]. Both of them lack a bZIP domain but suppress 5’ LTR-driven transcription, and they show different subcellular localizations: APH-3 is found in both nucleus and cytoplasm, whereas APH-4 only in the nucleus [91]. Again, the simian counterparts of HTLV-2, 3 and 4 (called STLV-2, 3, and 4) also encode antisense proteins [95].

Finally, recent studies have shown that the Bovine Leukemia Virus (BLV) expresses two antisense transcripts (AS1 and AS2) both in leukemic and in asymptomatic samples [96] (Table 1). Expression of these transcripts is under the control of IRF binding sites and E-box in the 3’ LTR [96]. Both transcripts initiate in the U5 region of the 3’ LTR and are spliced. AS1 undergoes alternative polyadenylation and generates two forms: a short one (AS1-S) of ~ 600nt, and a long one (AS1-L) of ~ 2200 nt. The AS2 transcript is present in a single form of ~ 400 nt. While AS1 and AS2 include a 264-nt ORF, their actual protein coding potential is unlikely based on analysis using Coding Potential Assessment Tools and also due to their nuclear localization, which suggests a predominantly regulatory role [96]. Moreover, ablation of BLV noncoding antisense transcripts has been shown to reduce proliferation of infected cells, suggesting that these molecules play a role in the leukemic process [97].

In summary, Deltaretroviruses – just like Lentiviruses – express antisense transcripts from the 3’ LTR. In the case of HTLVs, antisense transcripts are bifunctional molecules with both protein-coding and noncoding roles. However, in the case of BLV, the antisense transcripts have only regulatory, noncoding activity.

*Gammaretroviruses.* To date, the expression of antisense transcripts or proteins has not been reported for any members of this *genus* of simple retroviruses. However, two studies have shown that the LTR of the murine leukemia virus (MLV) is capable of driving bidirectional transcription (Table 1). Studies by the Pedersen group showed that in T- and B-cell lymphomas, the MLV 5’ LTR can direct antisense transcription from multiple start sites within the U3 region [28, 98]. These 5’ LTR-driven transcripts extend into neighboring host genome and produce chimeric RNA molecules expressing proto-oncogenes such as *Bach2* and *Jdp2*, which cause cell transformation. The authors confirmed these results using a knock-in mouse model homozygous for a single copy of the MLV LTR inserted in a genomic region that activates *Nras* in B-cell lymphomas [28]. Subsequently, Arpin-André et al. reported molecular studies that confirmed the ability of the MLV LTR to drive bidirectional transcription [26]. These studies also showed that in the absence of their respective viral transactivators, the LTRs of HIV-1 and HTLV-1 showed much weaker sense transcription than the LTR of MLV (which does not encode a transactivator). However, the three retroviral LTRs displayed similar antisense transcription activity [26].

These studies demonstrate that, although simple retroviruses have not been shown to express antisense transcripts or proteins, their LTRs do have properties of bidirectional promoters, and therefore are potentially capable of driving antisense transcription.

### Antisense transcription in endogenous retroviruses

As discussed above, vertebrate genomes contain a high proportion of transposable elements, many of which have retroviral origin [3, 99]. Indeed, sequencing of the human genome has revealed that ~ 8% is occupied by various forms of endogenous retroviruses (ERV), including full-length viral genomes, partial genomes, and as many as 25,000 isolated (solo) LTRs [6, 100]. These are sequences that originated from integration of now-extinct exogenous retroviruses over millions of years [2, 3]. Ancestral retroviruses may have been capable of infecting cells of the germ line, which ensured transmission to the offspring. The mechanism through which ERV formed multiple integration sites is similar to the one used by exogenous retroviruses, but it is confined to the same cell. It entails expression of the genomic RNA, reverse transcription into dsDNA, and then integration into a different site of the host genome.

To date, there is no evidence that endogenous retroviruses encode antisense proteins in the proviral minus strand in a manner similar to primate T-lymphotropic viruses (bZIP proteins) and group M HIV-1 strains (ASP). However, multiple studies have demonstrated that ERVs, much like exogenous retroviruses, express antisense transcripts (Table 1). Analysis of HERV-K subtype HML-2 expression in prostate cancer cells revealed that this ERV is capable of both sense and antisense transcription [101]. The same report also provided evidence that solo LTRs are transcribed in both directions [101]. A similar study focusing on a model of mammary epithelial cell transformation detected expression of HERV-K (HML-2) in both directions with the majority of transcription being in the antisense orientation [102]. A more recent study showed that differentiation of monocytes into macrophages in response to ionizing radiation is associated with reactivation of > 600 retroelements, especially several clades of HERVs [103]. In the case of HERVs, this involved induction of both sense and antisense transcription, with the latter expressed at 3- to 5-fold higher levels than the former [103]. Expression of HERVs has also been investigated in the context of HIV-1 infection [104, 105]. These studies found the presence of HERV-K (HML-2) RNA and proteins in plasma of PLWH, and some of the RNA sequences derived from newly discovered proviruses [105]. Also, these RNA molecules had been expressed from intact proviruses as well as proviruses lacking the 5’ LTR, suggesting that they originated from antisense transcription [105].

There is also ample evidence that ERV LTRs are able to drive bidirectional transcription from studies that investigated the expression of ERV-host chimeric transcripts (for a comprehensive review see [106]. For instance, in the Down Syndrome Critical Region (DSCR), the HERV1 LTR acts as a bidirectional promoter leading to the expression of *DSCR4* and *DSCR8* transcripts [30]. In another example, in K562 cells the bidirectionally active HERV9 LTR has been shown to direct the expression of an isoform of the C*ADM2* transcript that is shorter than in other cell types [106]. Evidence of bidirectional LTR activity has also been shown in mice [107]. The murine ERV-L (MuERV-L) and intracisternal A particle (IAP) are activated in the preimplantation mouse embryo, and sense and antisense transcripts are expressed at the 2- and 8-cell stage embryo [107]. Interestingly, host-mediated RNA interference limits the expression of these two retroelements as a way to protect genomic integrity at this early stage of development [107].

Therefore, the LTRs of endogenous retroviruses function as bidirectional promoters and direct antisense transcription despite the absence of antisense open reading frames and even in case of partial or complete deletion of the proviral genome (solo LTR). This ability is in line with what has been discussed above in regard to exogenous retroviruses, and it appears to be a feature of all retroelements.

### Origin of bifunctional transcripts

Bifunctional RNAs (also known as “coding-noncoding RNAs”, or cncRNAs) are defined as transcripts that have both protein-coding and noncoding (regulatory) activities. They have been described in all living organisms, including bacteria (*RNAIII* in *S. aureus*, *SR1* in *B. subtilis*, and *SgrS* in *E. coli*), plants (*ENOD40* in *M. trunculata*), insects (*oskar* in *D. melanogaster*), and vertebrates (*squint* in *D. rerio*, *vegt* in *X. levis*, *Ube3a* in *R. norvegicus*, *Sra1* in *M. musculus*) [108, 109]. Humans are no exception, and many examples of bifunctional transcripts have been also described in *Homo sapiens* [110].

Studies conducted over the last few years have shown that ~ 40% of transcripts considered to be “true” long noncoding RNAs (functional transcripts without protein coding activities) are associated with ribosomes in the cytoplasm and may be translated [111–113]. Some lncRNAs have actually been shown to contain short open reading frames that are translated into peptides with biological roles. For instance, the lncRNAs LINC00948, LINC00116, and LOC100507537 express the micropeptides Myoregulin (46 aa), Mitoregulin (56 aa) and Dwarf (34 aa) [114–116]. A more recent study identified 129 additional examples of lncRNAs that contain small ORF translated into short polypeptides with biological function [117]. At the other end of the spectrum, we find transcripts with known protein-coding function and that also have regulatory roles. Two examples are the mRNAs *SRA*, which interacts with and activates the transcription factor, MyoD [118], and *p53*, which interacts with and inactivates the E3 ubiquitin-protein ligase mdm2 [119].

The antisense transcripts *HBZ* of HTLV-1 and *Ast* of HIV-1 have both protein-coding and non-coding activities [50, 68]. Did these two bifunctional transcripts originate as lncRNAs that later developed protein-coding capacity or, vice versa, as mRNAs that have acquired regulatory activity? Which is the ancestral and which is the acquired function? While this question remains to be addressed conclusively, two lines of evidence may indicate a possible answer.

First, as discussed above, our current understanding indicates that antisense transcription is a common feature among retroviruses – both endogenous and exogenous, and simple and complex (Table 1). In many cases these antisense transcripts are not translated into a protein product. We also have evidence that retroviral LTRs have an intrinsic ability to direct bidirectional transcription even in the case of ERVs that lack antisense ORFs or in the case of solo LTRs.

Second, a recent study from the Matsuoka group has demonstrated that even antisense transcripts with protein-coding capacity, such as *HBZ* and *Ast*, display a predominantly nuclear distribution due to inefficient polyadenylation [43]. In their study, the authors presented evidence that this is not the consequence of weak polyadenylation signals, but more likely of the weak native promoters in the 3’ LTRs [43].

Thus, the evidence that expression of antisense transcripts is phylogenetically more widespread among retroviruses than expression of antisense proteins along with the predominantly nuclear distribution of bifunctional antisense transcripts that favors regulatory over protein-coding roles suggest that retroviral antisense transcripts may have originated as lncRNAs, which in some cases have acquired the ability to function as mRNAs. Confirmation of this hypothesis requires further studies, including a direct demonstration that non-pandemic HIV-1 strains and SIV strains – which do not encode antisense proteins – are capable of antisense transcription.

### Regulatory activity of natural antisense transcripts

Antisense transcripts can modulate the expression of their cognate protein-coding sense genes [120]. Most often, antisense transcripts inhibit expression of the sense gene, but cases have been reported where they increase expression of the sense transcript by protecting it from nuclease degradation [121]. The regulatory function of antisense transcripts is mediated by the RNA molecule itself and/or by the act of antisense transcription itself. This can occur at various, non-mutually exclusive steps during the expression of the sense RNA molecule: initiation, processing, transport, stability, and translation [122]. For comprehensive reviews, see [47, 123–125].

Antisense RNAs inhibit transcription of their cognate sense gene through at least four mechanisms (collectively, transcriptional interference or TI) [125]:

- *promoter competition*: sense and antisense RNA are expressed from a bidirectional promoter. Assembly of the transcriptional machinery expressing the antisense RNA blocks or prevents expression of the sense RNA.
- *binding site occlusion*: the RNA polymerase complex expressing the antisense transcript prevents chromatin access to transcription factors required for expression of the sense transcript.
- *RNA polymerase collision*: the antisense transcriptional machinery displaces the machinery assembled onto the promoter of the sense transcript’s (‘sitting duck’), or it stalls the incoming sense transcriptional machinery (‘roadblock’).
- *epigenetic silencing*: the antisense transcript recruits chromatin remodeling factors at the promoter region of the sense transcript, leading to a closed chromatin status and transcriptional repression. We showed this to be one of the mechanisms through which the HIV-1 antisense transcript *Ast* promotes silencing of the proviral 5’ LTR and viral latency [38].

Antisense transcripts can also regulate the expression of their paired sense transcript post-transcriptionally through three mechanisms involving formation of double stranded RNA complexes:

- *RNA masking*: formation of a sense-antisense duplex that blocks the interaction of the sense transcript with factors (proteins and miRNAs) that regulate its splicing, stability, transport, and translation [124, 125].
- *RNA interference*: recognition of the RNA duplex by Dicer, with subsequent cleavage and formation of ‘endo-siRNAs’ [125]. Alternatively, the RNA duplex is recognized by Protein Kinase R (PKR), which undergoes dimerization and autophosphorylation, suppresses protein expression, and ultimately triggers IFNα/β innate immune responses.
- *RNA editing*: recognition of duplex RNA by members of the ADAR protein family that deaminate adenosine residues into inosine, which results in amino acid changes [124].

As discussed above, antisense transcription has been documented among many *genera* of endogenous and exogenous retroviruses even in the absence of protein-coding function (Table 1). What selective advantage does the noncoding function of antisense transcripts afford to the virus? What purpose does it serve in the virus life cycle?

Bimodal (ON-OFF) gene expression requires the establishment of thresholds that ensure stability of the system in the two states (bistability) and that regulate the switch between the two states. In protein-based regulatory systems, threshold generation is achieved through cooperativity [126–128]. For example, in the case of HIV-1, the choice between ON (viral expression) and OFF states (viral latency) is thought to be regulated entirely by the HIV-1 transactivator Tat through a mechanism of positive-feedback loop. However, since Tat is a monomeric transactivator that does not bind the TAR sequence cooperatively, classical models would predict an instability of the OFF state (viral latency) [128]. Moreover, Tat is not sequestered or neutralized by natural inhibitors, and thus it is not possible to invoke a threshold-linear mechanism of transcriptional silencing to achieve and stabilize viral latency [129, 130]. Given this evidence, how does HIV-1 achieve stable proviral latency?

Ultrasensitivity is another mechanism of threshold generation that governs the switch between two alternative transcription states [131–133]. It stabilizes the OFF state by ignoring weak stimuli, but it allows a rapid, sigmoid-like switch to the ON state in response to strong signals [133]. Therefore, in systems where the protein transactivator lacks cooperativity or inhibitors (such as the HIV-1 transactivator Tat), ultrasensitive responses contribute to achieve bistability.

**Fig. 3:**
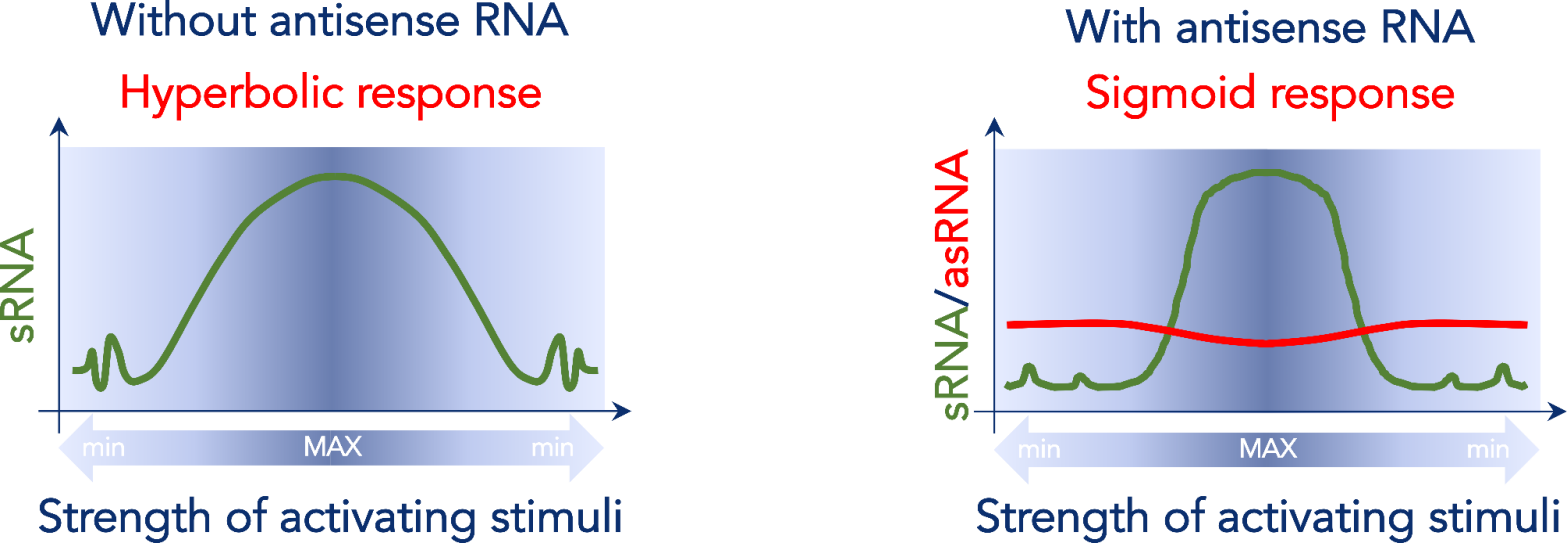
Dynamics of ON/OFF sense transcription in the absence (left) and presence (right) of cognate regulatory antisense transcript. Left panel (in the absence of regulatory antisense RNA): at low levels of activating stimuli, OFF state of sense transcription is unstable and noisy, and it is induced stochastically in response to transient fluctuations of the signal. As activating stimuli strengthen, sense transcription slowly increases and gradually reaches maximum levels along a hyperbolic curve (ON). Similarly, return to baseline levels of sense transcription occurs gradually with the waning of the activating stimuli. Right panel (in the presence of regulatory antisense RNA): at low levels of activating stimuli (OFF), the antisense transcript buffers spurious activating stimuli, thus dampening noisy expression of sense transcripts and achieving a stable OFF state. When activating stimuli are sufficiently strong to overcome the threshold set by the antisense RNA, sense transcription is induced and rapidly reaches maximum levels along a sigmoid curve (ON). As activating stimuli weaken below the ultrasensitive threshold, expression of sense transcripts rapidly drops to baseline levels following an inverse sigmoid curve and returns to a stable OFF state

A number of reports have shown that some antisense transcripts can establish an ultrasensitive ON-OFF switch regulating the expression of their cognate sense transcript (Fig. 3) [123]. When antisense transcription precedes that of the sense transcript, the antisense RNA buffers weak stimuli (transient, spurious signals) and sets a threshold dampening stochastic variations in the expression of sense RNA [134, 135]. Moreover, repression of sense transcription is more robust in the case of antisense transcripts that exert their regulatory role both transcriptionally and post-transcriptionally [136]. However, when activating stimuli are sufficiently strong to overcome the threshold set by the antisense transcript, then sense transcription is switched on reaching maximal levels very rapidly (sigmoid curve). In the ON state, the continued presence of antisense RNAs can lead to transcriptional bursting of the sense gene and increase cell-to-cell variability [137]. Finally, when the activating stimuli decline and cease, antisense RNAs contribute to a faster return to baseline levels of the sense RNA [137]. Therefore, antisense transcripts establish ultrasensitive thresholds that dampen stochastic expression of the sense gene in conditions of sub-optimal signals, allow rapid expression of the sense gene when activating stimuli are sufficiently strong, and promote a faster return to baseline levels when activating stimuli subside. It also worth noting that these thresholds are established independently for each locus or cell, which increases variability and adaptation [138].

In the case of retroviruses, it is intriguing to speculate that antisense transcription might provide a mechanism to regulate sense gene expression through the establishment of ultrasensitive thresholds. This strategy allows the virus to stabilize the latency phase, and to control the switch to and from replication. Moreover, this can be achieved without the need to encode yet another open reading frame in small and densely packed viral genomes. Importantly, HIV-1 and HTLV-1 express antisense transcript relying primarily on ubiquitous cellular transcription factors independently of the viral transactivators, Tax and Tat [23–25, 27], which ensures constitutive expression. Studies on the non-protein coding function of HIV-1 antisense transcripts seem to support this interpretation, and they suggest that HIV-1 antisense transcripts suppress sense transcription via epigenetic silencing of the 5’ LTR [37, 38, 46].

This regulatory mechanism affords the virus a greater control over its destiny because it offers the ability to choose between replication and latency in response to the cell environment. Under conditions that are not ideal for production of viral progeny, expression of viral proteins would be a risk without benefit, because it would unnecessarily expose the infected cell to recognition and elimination by the immune system. At the same time, as we discussed above, positive-feedback loops where the viral transactivator (e.g., HIV-1 Tat) lacks cooperativity or natural inhibitors cannot ensure stability of viral latency (OFF state). Using antisense transcription as a means to establish an ultrasensitive threshold allows the virus to stabilize latency when the cell does not provide the ideal environment for viral replication, thus avoiding unnecessary immune recognition, ensuring viral persistence, and increasing the chances for spread.

## Conclusions

Antisense transcription is a property of many endogenous and exogenous retroviruses. This is not only reflected in the expression of antisense RNA molecules, but also in the intrinsic ability of retroviral LTRs to direct bidirectional transcription. Moreover, retroviruses are capable of antisense transcription even in the absence of open reading frames encoded in the minus strand of the proviral genome. The broad phylogenetic distribution of antisense transcription across the *Retroviridae* family suggests important roles in the virus lifecycle that precede and go beyond protein-coding functions. Among other plausible roles, antisense transcripts may have evolved as a strategy to stabilize viral latency when the cell environment does not offer optimal conditions to sustain viral replication, while at the same time allowing rapid reversal of latency and maximal viral expression in response to strong activating stimuli.

## Data Availability

Not applicable.

## List of abbreviations

ASP: antisense protein
BIV: bovine immunodeficiency virus
BLV: bovine leukemia virus
cncRNA: coding-noncoding RNA
FIV: feline immunodeficiency virus
SIV: simian immunodeficiency virus
ERV: endogenous retrovirus
HBZ: HTLV-1 bZIP
HERV: human endogeneous retrovirus
IAP: intracisternal A particle
lncRNA: long noncoding RNA
LTR: Long Terminal Repeat
HIV-1: Human Immunodeficiency Virus 1
HTLV-1/2/3/4: Human T-lymphotropic Virus 1/2/3/4
MLV: murine leukemia virus
ncRNA: noncoding RNA
NSP: negative sense promoter
PLWH: people living with HIV-1
PSP: positive sense promoter
STLV: simian T-lymphotropic virus
